# Evolution of form in metal–organic frameworks

**DOI:** 10.1038/ncomms14070

**Published:** 2017-01-04

**Authors:** Jiyoung Lee, Ja Hun Kwak, Wonyoung Choe

**Affiliations:** 1 Department of Chemistry, Ulsan National Institute of Science and Technology, Ulsan 44919, Republic of Korea; 2 Department of Chemical Engineering, Ulsan National Institute of Science and Technology, Ulsan 44919, Republic of Korea

## Abstract

Self-assembly has proven to be a widely successful synthetic strategy for functional materials, especially for metal–organic materials (MOMs), an emerging class of porous materials consisting of metal–organic frameworks (MOFs) and metal–organic polyhedra (MOPs). However, there are areas in MOM synthesis in which such self-assembly has not been fully utilized, such as controlling the interior of MOM crystals. Here we demonstrate sequential self-assembly strategy for synthesizing various forms of MOM crystals, including double-shell hollow MOMs, based on single-crystal to single-crystal transformation from MOP to MOF. Moreover, this synthetic strategy also yields other forms, such as solid, core-shell, double and triple matryoshka, and single-shell hollow MOMs, thereby exhibiting form evolution in MOMs. We anticipate that this synthetic approach might open up a new direction for the development of diverse forms in MOMs, with highly advanced areas such as sequential drug delivery/release and heterogeneous cascade catalysis targeted in the foreseeable future.

## Abstract (web summary)

Self-assembly is a strategy for making metal-organic materials but controlling the interior of metal-organic crystals remains challenging. Here, the authors report a sequential self-assembly process for synthesizing various interior morphologies of metal-organic crystal demonstrating evolution of form.

For the past few decades, a main theme of materials chemistry has been to control the exterior and interior of solid-state materials to meet the rising demand for new, high-performance functional materials^1^,^2^,^3^,^4^,^5^,^6^. In particular, controlling the interior of the particles and thereby transforming solid particles into more sophisticated forms, such as core-shell, hollow, matryoshka (for example, Russian doll), yolk-shell and multi-shell hollow particles, has been an important synthetic challenge for materials chemists because these exotic materials could exhibit advanced functional properties in catalysis^7^,^8^,^9^,^10^, chemical sensing^11^,^12^,^13^, energy^14^,^15^,^16^,^17^ and biomedical applications^18^,^19^,^20^,^21^ (Fig. 1). Recently, similar synthetic efforts have been applied to metal–organic frameworks (MOFs), an emerging class of porous materials^22^,^23^,^24^,^25^,^26^,^27^,^28^,^29^,^30^,^31^,^32^,^33^,^34^,^35^,^36^. However, unlike other micro-/nanostructures reported in metal/metal oxide systems, those in MOFs are still in their infancy, as evidenced by the fact that the vast majority of these materials have hollow forms, an early stage of form evolution (Fig. 1)^37^. The synthetic strategies identified for hollow MOFs include templating methods (using polystyrene beads^25^,^26^, emulsion droplets^27^, CO_2_ bubbles^28^, MOFs^29^ and metal–organic polyhedron (MOP) crystals^30^), interfacial growing methods^31^, spray-drying techniques^32^ and surface-driven mechanisms^33^. A recent notable example from Lah group utilizes MOP crystals as a sacrificial template to synthesize hollow MOFs^30^. Despite these efforts, making further sophisticated forms in MOFs is significantly hampered by a lack of rational synthetic strategies.

Here, we first demonstrate a synthetic strategy for double-shell hollow MOF via sequential self-assembly. The sequential steps involved in creating double-shell hollow MOF are shown in Fig. 2: (1) single-crystal to single-crystal transformation from MOP to MOF through postsynthetic linker insertion (I-a), (2) epitaxial growth of MOP on the MOF surface (I-b), (3) insertion of another linker to form double to triple-matryoshka metal–organic materials (MOMs) (I-c), and finally (4) elimination of MOP by chemical etching (I-d). Through this stepwise synthetic procedure, we successfully complete form evolution from a parent MOP to various MOMs, including solid, core-shell, double and triple matryoshka, and hollow single- and double-shell structures.

## Results

### Synthesis of cuboctahedron MOP and its solid-state structure

Our journey to form evolution in MOMs begins with cuboctahedron MOPs (hereafter, **cuo**-MOPs). These MOPs are composed of 12 Cu_2_(COO)_4_ paddlewheel nodes and 24 5-R-1,3-benzenedicarboxylic acid (R-mBDC, R=H, OH, NO_2_, SO_3_^−^ and so on)^38^,^39^ (Fig. 3a), and some of these MOPs prefer face-centered cubic (fcc) packing. As reported by Eddaudi and Lah^40^,^41^, there is a striking structural similarity between such fcc-packed cuo-MOPs and their corresponding linker-inserted MOFs with **ubt** topology (Fig. 3b,c). The **ubt**-MOFs can be obtained by connecting the axial position of paddlewheel nodes in **cuo**-MOPs with linear linkers, such as 1,4-diazabicyclo[2.2.2]octane (dabco) and 4,4′-bipyridyl (bpy) through conventional solvothermal synthesis (Fig. 3c). For example, Chun *et al*. reported **ubt**-MOFs by connecting the twelve Zn(II) paddlewheel nodes of a MOP cage with their neighbouring MOPs via dabco,^42^ while Wang group synthesized Cu(II) paddlewheel-based ubt-MOFs using amino-functionalized cuo-MOPs and bpy linkers^43^. Although there is no report demonstrating single-crystal to single-crystal transformation from **cuo**-MOP to **ubt**-MOF, we hypothesize that by careful choice of **cuo**-MOP, a structural transformation from fcc-packed **cuo**-MOP to **ubt**-MOF might be possible via postsynthetic insertion of dabco linkers^44^,^45^,^46^.

To connect adjacent **cuo**-MOPs with dabco linkers, the ideal Cu–Cu distance between the two paddlewheel nodes is estimated as 6.6–7.5 Å based on a Cambridge Structure Database search (Supplementary Table 1). A new member of the fcc-packed **cuo**-MOP family, UMOM-1 [Cu_24_(OH-mBDC)_24_(DMSO)_8_(H_2_O)_16_] has been synthesized and characterized by X-ray single-crystal diffraction, exhibiting a Cu–Cu distance of 7.5–8.2 Å, which is slightly longer than the ideal distance for dabco insertion (Supplementary Figs 1–4 and Supplementary Data 1) due to a slight rotation of the MOP cages. Although the cages are not perfectly aligned for dabco insertion, the packing of the MOP cages are close to ideal fcc packing, which is a desired feature for the planned structural transformation (Fig. 3d).

### Single-crystal to single-crystal transformation of MOP to MOF

When we soaked the crystals of UMOM-1 in a N,N′-dimethylformamide (DMF)/dimethyl sulfoxide (DMSO) solution (v/v=1:1) containing dabco, after 12 h, we noticed a crystal colour change from blue to green without any visual cracks in the optical microscopic images (Fig. 3e and Supplementary Fig. 5). Subsequent X-ray single-crystal analysis confirmed that this is indeed a single-crystal to single-crystal transformation from MOP (UMOM-1) to MOF (UMOM-2) (Supplementary Fig. 6, Supplementary Table 2 and Supplementary Data 2). When we compared the structures of UMOM-1 and UMOM-2, the space group was changed from *I*4*/m* to *Fm*-3*m* with 0.87% cell volume reduction (Supplementary Tables 3 and 4). In UMOM-2, as expected, the cofacial paddlewheel nodes are connected by dabco with a Cu–Cu distance of 7.0 Å (Supplementary Figs 7 and 8), which is a significant decrease from those of 7.5 and 8.2 Å in the parent MOP, UMOM-1. ^1^H NMR spectra was obtained from digested solution of UMOM-2 to confirm the insertion of dabco (Supplementary Fig. 9). The ratio of OH-mBDC to dabco is 4.00:1.02, which agrees well with the ratio found in the X-ray single-crystal analysis.

When the structural transformation from UMOM-1 to UMOM-2 was monitored by X-ray synchrotron powder diffraction (Fig. 3f and Supplementary Fig. 10) at the Pohang Accelerator Laboratory (PAL) in the Republic of Korea, noticeable 2*θ* changes could be seen at 9.6°, 9.8° and 10.7°, which correspond to (2–13), (222) and (3–12) reflections, respectively, for the parent UMOM-1. During the transformation, the intensity of the (2–13) reflection decreased, while the intensity of the (222) reflection increased with a slight shift toward higher 2*θ* values. Owing to cell parameter changes, the (3–12) reflection shifted to lower 2*θ* values. These results clearly indicate that there is a progressive change from UMOM-1 to UMOM-2 during the linker insertion reaction.

To propose a plausible mechanism for structural change, the transformation can be simulated using both the rotation and translation of MOP cages (Supplementary Fig. 11). When compared with the experimental X-ray synchrotron powder diffraction patterns, a 8.9° clockwise rotation can occur along the c-axis, together with a 0.18 Å translation inward (Supplementary Figs 12 and 13). From these results, we conclude that both rotation and translation of MOP cages are necessary to accommodate dabco molecules inside UMOM-1.

The resulting MOF, UMOM-2, is a porous material, as confirmed by the N_2_ sorption isotherm at 77 K (Supplementary Figs 14 and 15) with Brunauer–Emmett–Teller (BET) and Langmuir surface areas of 2,540 and 2,820 m^2^ g^−1^, respectively. The calculated surface area (Connolly surface) is 3,030 m^2^ g^−1^, as determined with a 1.4 Å van der Waals scale factor and 1.84 Å Connolly radius using Material Studio. The pore size distribution of UMOM-2 from the N_2_ isotherm using the oxide surface cylindrical model shows three different types of pores: 10.9, 15.6 and 18.8 Å, corresponding to the pore diameters of three types of cages (truncated tetrahedron, cuboctahedron and truncated octahedron, respectively, as identified in single-crystal structures) (Supplementary Fig. 16). CO_2_ adsorption-desorption isotherm was also obtained, and the maximum adsorbed amount of CO_2_ is 3.4 mmol g^−1^ at 298 K and 6.8 mmol g^−1^ at 273 K (Supplementary Fig. 17). These values are comparable to those of rht-MOF-7 (4.0 mmol g^−1^ at 298 K and 6.5 mmol g^−1^ at 273 K)^47^, also known as Cu-TDPAT^48^, a leading MOF for CO_2_ capture with **rht** topology, which has structural similarity with **ubt** topology^41^,^49^. The X-ray powder diffraction patterns show that the sample does not undergo any phase changes after activation (Supplementary Fig. 18). The peak positions of as-synthesized UMOM-2 and activated UMOM-2 match exactly. Thermogravimetric analysis shows that UMOM-2 is thermally stable up to ∼250 °C (Supplementary Fig. 19).

The kinetic profile of the transformation procedure was obtained using ^1^H NMR analysis (Supplementary Fig. 20). The kinetic profile shows that the process becomes slower over time.

### Synthesis of single-crystal single-shell hollow MOF

**Figure 2:**
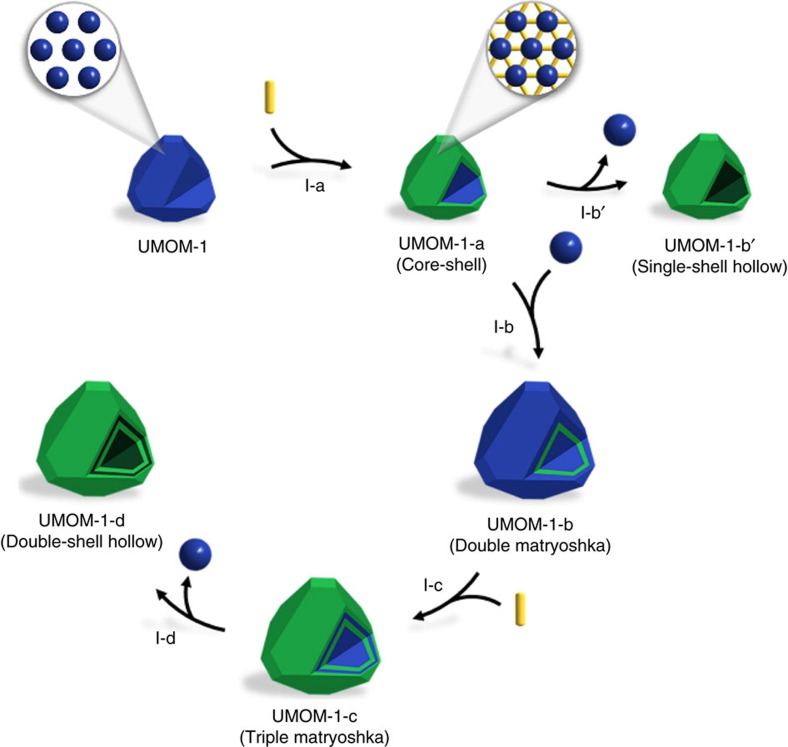
Schematic illustration of form evolution. The process of synthesizing various forms of MOMs, including UMOM-1-a, UMOM-1-b, UMOM-1-b′, UMOM-1-c and UMOM-1-d from UMOM-1. Stage I-a, UMOM-1 (MOP)→UMOM-1-a (core-shell) by partial postsynthetic linker insertion. Stage I-b′, UMOM-1-a→UMOM-1-b′ (single-shell hollow) by etching process. Stage I-b, UMOM-1-a→UMOM-1-b (double matryoshka) by epitaxial growth of UMOM-1 on the surface of UMOM-1-a. Stage I-c, UMOM-1-b→UMOM-1-c (triple matryoshka) by partial postsynthetic linker insertion. Stage I-d, UMOM-1-c→UMOM-1-d (double-shell hollow) by etching process. Blue sphere represents **cuo**-MOP and yellow rod represents dabco linker.

**Figure 3:**
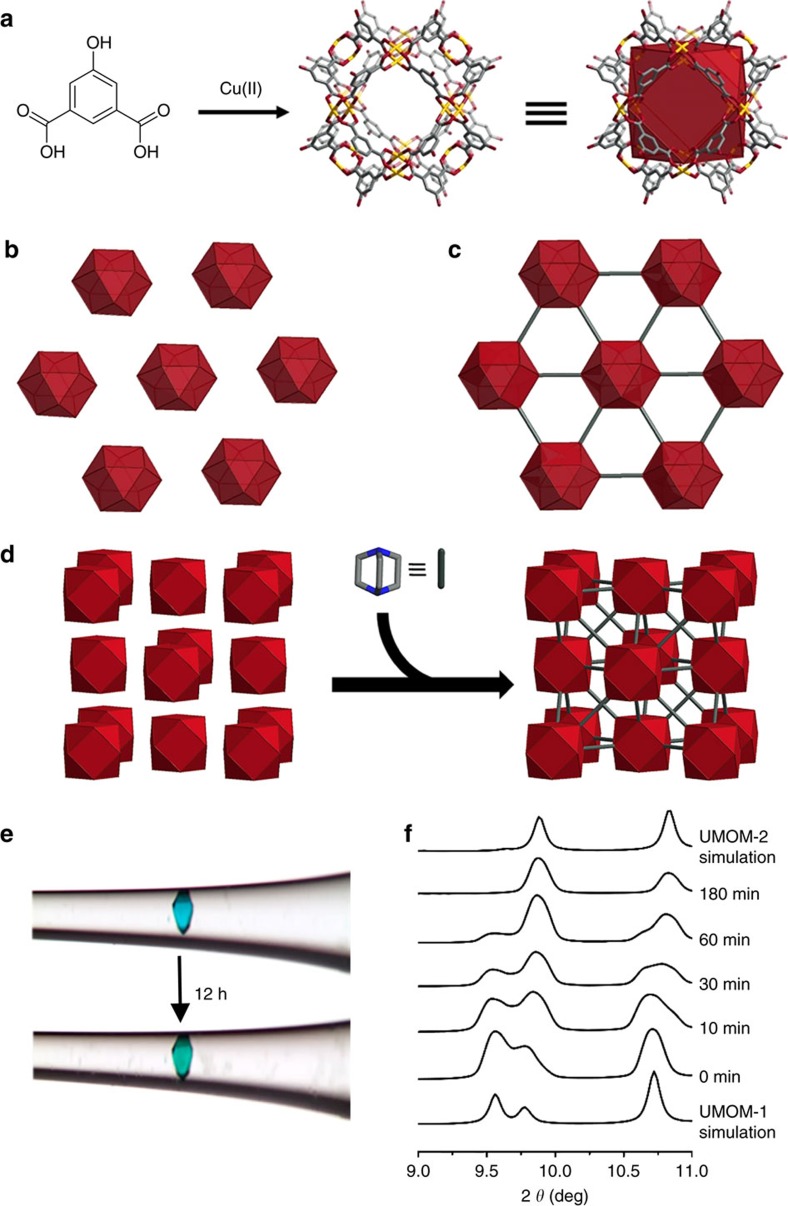
Structural similarity between ubt-MOF and fcc-packed cuo-MOP. (**a**) The building blocks for construction of **cuo**-MOP; OH-mBDC as an organic linker and Cu_2_(COO)_4_ paddlewheel as a metal node. Cu, orange; C, grey; O, red; all hydrogen and solvents on the Cu(II) paddlewheel are omitted for clarity. Perspective view of the 3-fold axis of (**b**) fcc-packed **cuo**-MOP (UMOM-1) and (**c**) **ubt**-MOF (UMOM-2). Grey line represents linear dabco. (**d**) Scheme of single-crystal to single-crystal transformation from UMOM-1 to UMOM-2. (**e**) Photographs of the single crystal before (top of panel, UMOM-1) and after (bottom of panel, UMOM-2) structural transformation. (**f**) Monitoring the structural transformation using X-ray synchrotron powder diffraction. The crystals were collected by quenching during linker insertion reaction time at 0, 10, 30, 60 and 180 min.

**Figure 4:**
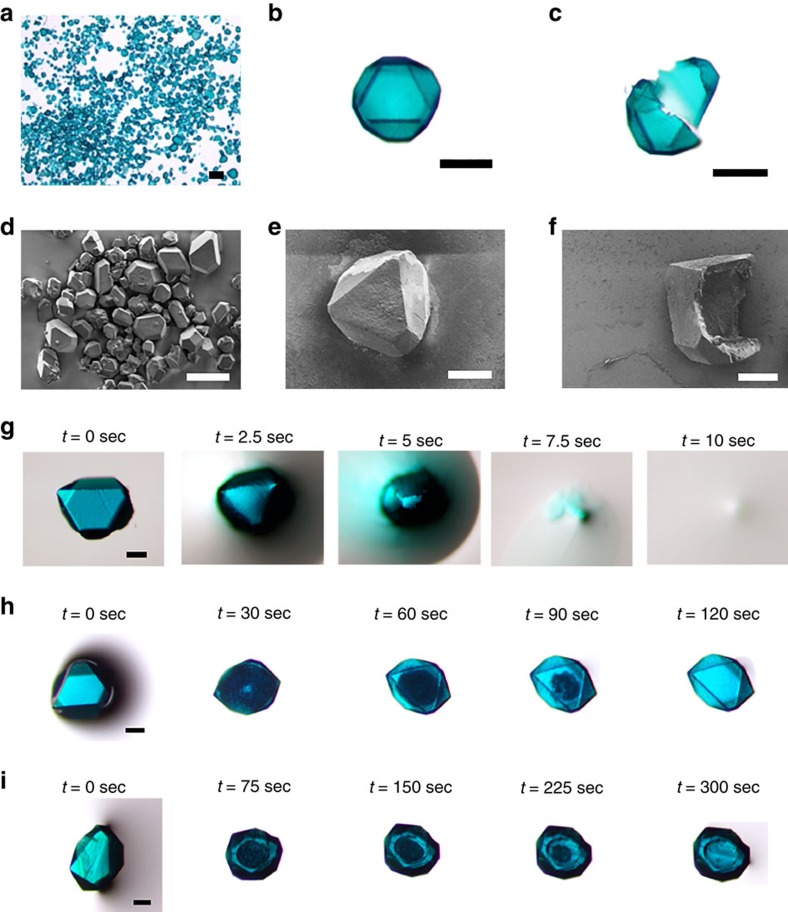
Microscopic images of single-shell hollow UMOM-1-b′. (**a**) Optical microscopic image of overview of UMOM-1-b′. Scale bar, 500 μm. (**b**,**c**) Optical microscopic images of single crystal of UMOM-1-b′ before and after crack using needle tip. Scale bar, 200 μm. (**d**) SEM image of overview of UMOM-1-b′. Scale bar, 100 μm. (**e**) SEM image of single crystal of UMOM-1-b′. Scale bar, 50 μm. (**f**) SEM image of cracked single crystal of UMOM-1-b′. Scale bar, 50 μm. Time course images: (**g**) Single crystal of UMOM-1 in methanol. Single crystal of UMOM-1-b′ in methanol with different linker insertion time (**h**) 10 min, and (**i**) 90 min. Scale bar, 200 μm (**g**–**i**). Linker insertion time is 30 min (**a**–**c**), and 10 min (**d**–**f**).

**Figure 5:**
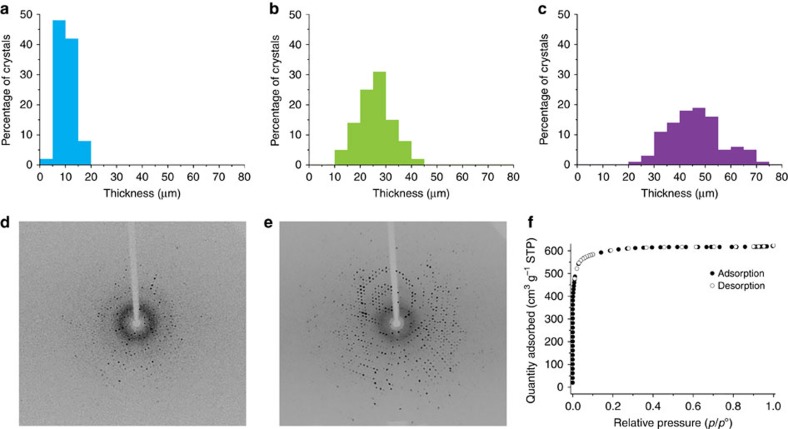
Characteristics of single-shell hollow UMOM-1-b′. Shell thickness distribution of UMOM-1-b′ depending on the different linker insertion time: (**a**) 30 min, (**b**) 90 min and (**c**) 120 min. The distribution data are collected with 100 crystals by using optical microscope. Single-crystal X-ray diffraction patterns of UMOM-1-b′ with different linker insertion time: (**d**) 10 min, and (**e**) 90 min. (**f**) N_2_ sorption isotherm of UMOM-1-b′, resulting of 30 min linker insertion reaction.

While synthesizing UMOM-2, instead of making a solid MOF, we quenched the reaction during linker insertion, resulting in a core-shell system of MOF@MOP (from shell to core), which we call UMOM-1-a (Fig. 2). Such hybrid core-shell systems between MOP and MOF have not been established previously. Interestingly, we note that UMOM-2 is not soluble in methanol while UMOM-1 is. This is due to a drastic solubility change as a result of forming an extended solid. This drastic difference in solubility makes methanol an ideal solvent for chemical etching to create a hollow crystal. In other words, methanol can dissolve only MOP in the core-shell system of UMOM-1-a, leaving MOF in the outer shell untouched. When we treated UMOM-1-a with methanol, single-shell hollow crystals formed (UMOM-1-b′). Figure 4 and Supplementary Movies 1 and 2 clearly show the formation of single-shell hollow crystals of UMOM-1-b′. Interestingly, the thickness of the shell could be controlled by the reaction time (t) for linker insertion (Fig. 5a–c). The average shell thickness is 10, 26 and 40 μm at *t*=30, 90, and 120 min, respectively. Optical microscopic images and scanning electron microscope (SEM) images clearly show the hollow interior of UMOM-1-b′ (Fig. 4a–f and Supplementary Fig. 21). Ultraviolet–visible absorbance spectra show that the copper ions are released from the crystals to the methanol solution during UMOM-1-b′ synthesis and etching reaction is completed in 200 min (Supplementary Figs 22 and 23). ^1^H NMR analysis of the digested methanol solution shows that only OH-mBDC of MOP is released but dabco is not (Supplementary Fig. 24). All these results pinpoint that dissolved MOP is released from the crystals during the etching process.

Surprisingly, single-shell UMOM-1-b′ is indeed single crystalline, as determined by X-ray single-crystal diffraction (Supplementary Figs 25 and 26 and Supplementary Data 3). We were able to collect several sets of single-crystal data for single-shell MOFs, and these data were successfully refined (Supplementary Table 5). In addition, the porosity of UMOM-1-b′ is maintained, as confirmed by N_2_ sorption, with the BET and Langmuir surface areas of 2,390 and 2,700 m^2^ g^−1^, respectively, which are slightly smaller than those for the solid MOF (Supplementary Figs 27 and 28). The pore size distribution of UMOM-1-b′ determined from the N_2_ isotherm using the oxide surface cylindrical model shows three different types of pores: 10.9, 15.6 and 18.8 Å, similar to those for the solid MOF counterpart (Supplementary Fig. 29).

### Synthesis of double-shell hollow MOF

**Figure 6:**
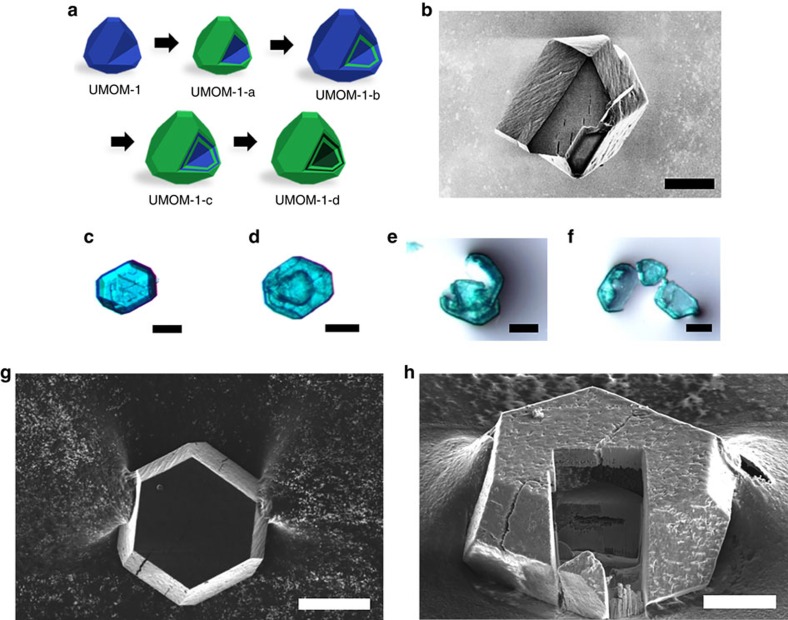
Microscopic images of double-shell hollow UMOM-1-d. (**a**) The evolution of form in MOFs: from UMOM-1, UMOM-1-a, UMOM-1-b, UMOM-1-c and finally to UMOM-1-d. (**b**) SEM image of single crystal which is in epitaxial growth process of UMOM-1 on the crystal surface of UMOM-1-a. Scale bar, 50 μm. Optical microscopic images of (**c**) UMOM-1-b, (**d**) UMOM-1-d and (**e**,**f**) broken crystal of UMOM-1-d. Scale bar, 100 μm (**c**–**f**). FIB-SEM images of single crystal of UMOM-1-d: (**g**) before the milling and (**h**) after milling with 52° rotation. Scale bar, 50 μm (**g**), 25 μm (**h**).

A double-shell hollow MOF was achieved by applying epitaxial growth of MOP to the crystal surface of core-shell UMOM-1-a. After soaking the crystals of UMOM-1-a in a DMSO/DMF mixture, a methanol solution containing Cu(II) and OH-mBDC was added to make double-matryoshka UMOM-1-b (MOP@MOF@MOP). The same procedure was repeated one more time to evenly grow MOP on the seed MOF crystals. We obtained a shell growth image from SEM analysis of the crystals during the epitaxial growth reaction (Fig. 6a). The optical microscopic images show the well-coated single-crystalline shell of UMOM-1-b (Fig. 6b). Triple-matryoshka UMOM-1-c (MOF@MOP@MOF@MOP) was obtained by quenching during linker insertion into UMOM-1-b. Finally, the double-shell UMOM-1-d was achieved using the same chemical etching method as used for single-shell UMOM-1-b′ (Fig. 6c). Optical microscopic images confirm the empty space between the two shells of UMOM-1-d (Fig. 6d,e). Focused ion beam-SEM (FIB-SEM) images also confirm the empty space after milling the surface of UMOM-1-d using a Ga ion beam (Supplementary Method). The cross-section of UMOM-1-d represents the two crystalline shells and the cavity of the double-shell hollow MOF (Fig. 6f,g), demonstrating the double-shell nature of UMOM-1-d.

Unlike UMOM-1-a, -b and -c, UMOM-1-d shows permanent porosity, as confirmed by the N_2_ sorption with the BET and Langmuir surface areas of 2,150 and 2,540 m^2^ g^−1^, respectively, which are comparable to those for the single-shell hollow MOF (Supplementary Fig. 30). Thermogravimetric analysis shows that UMOM-1-a, -b, -c and -d are thermally stable up to 230–250 °C (Supplementary Fig. 31).

## Discussion

In summary, we successfully demonstrated the first example of double-shell hollow MOF, UMOM-1-d, via sequential self-assembly, followed by self-disassembly. In addition, this strategy leads to complete form evolution from a parent MOP to various MOMs, including solid, core-shell, double and triple matryoshka, and hollow single- and double-shell forms. Another marked difference between these and other hollow MOF crystals is single crystallinity. While other hollow MOFs often consist of aggregates of small crystals, the hollow MOFs presented here are the first case where single crystallinity is demonstrated by X-ray single-crystal diffraction. We expect that such examples of extreme self-assembly might open up new avenues to various forms of MOFs for synthesizing single crystalline multi-shelled hollow MOF with hierarchical porous materials with trimodal (for example, micro-/meso-/macro porous structures) pore system and highly advanced applications such as sequential drug delivery/release and biomimetic cascade catalysis. A good case for the latter can be found in nature, as exemplified by native pyruvate dehydrogenase (PDH2), a multifunctional catalytic machine with an icosahedral double-hollow structure. PDH2 is composed of three different enzymes that are distributed individually in two shells^50^. This spatial separation is known to be crucial for the overall function of PDH2 and plays a key role in its catalytic behaviour. Mimicking such multi-component, macromolecular machines through surface modification or postsynthetic modification is a synthetic challenge for next-generation MOMs, and will further push the limit of self-assembly.

## Methods

### Materials

5-hydroxy-1,3-benzenedicarboxylic acid (OH-mBDC; TCI), 1,4-diazabicyclo [2.2.2] octane (dabco; Sigma-Aldrich), 4,4′-bipyridyl (bpy; TCI) and Cu(OAc)_2_·H_2_O (JUNSEI) were used without further purification. Methanol (MeOH), DMSO and DMF were obtained from JUNSEI, and N,N'-dimethylacetamide was obtained from TCI.

### Synthesis of UMOM-1

A MeOH (4.0 ml) solution of OH-mBDC (146.1 mg, 0.802 mmol) was mixed with a MeOH (12.0 ml) solution of Cu(OAc)_2_·H_2_O (160.0 mg, 0.801 mmol) in a capped vial (20 ml). After mixing, 2.5 ml of N,N'-dimethylacetamide and 1.5 ml of MeOH was added to this solution and then allowed the vial stand at room temperature. After 5 days, synthesized crystals were collected and dissolved in 20.0 ml of MeOH (solution-A). In total, 3.0 ml of solution-A was well mixed with 3.0 ml of DMSO/DMF solution (v/v=1:1) and then allowed the vial stand at room temperature. After 1 day, blue crystals were recrystallized.

### Synthesis of UMOM-2

The recrystallized UMOM-1 (∼30.0 mg) was immersed in the 0.18 M dabco solution with 10.0 ml of DMSO/DMF mixture (v/v=1:1) and then left to react at room temperature. After 1 day, green crystals were obtained.

### Synthesis of UMOM-1-a

The recrystallized UMOM-1 (∼30.0 mg) was immersed in 36.0 mM dabco solution with 5.0 ml of DMSO/DMF mixture (v/v=1:1) and then left to react at room temperature for time, *t* (10 min<*t*<180 min). The reacted crystals were rinsed with 5.0 ml pure DMSO/DMF mixture (v/v=1:1) for three times.

### Synthesis of UMOM-1-b′

UMOM-1-a was immersed in 5.0 ml of MeOH at room temperature. After 1 day, blue hollow crystals were collected.

### Synthesis of UMOM-1-b

In total, 2.0 ml of solution-A and 2.0 ml of DMSO/DMF solution (v/v=1:1) were carefully mixed and left to react at room temperature. After 4 h, blue crystals were recrystallized. After decanting the solution, 5.0 ml of 36.0 mM dabco solution was added into the recrystallized crystals and then left to react at room temperature for 10 min and then rinsed the with 5.0 ml pure DMSO/DMF mixture (v/v=1:1) for three times. After that, collected crystals were immersed in 3.0 ml of DMSO/DMF mixture (v/v=1:1) and 3.0 ml of solution-A/MeOH mixture (v/v=1:2) and then left to react at room temperature for 1 day. After decanting the solution, 3.0 ml of DMSO/DMF mixture (v/v=1:1) and 3.0 ml of solution-A/MeOH mixture (v/v=1:2) were added and well mixed with crystals then left to react at room temperature for 1 day.

### Synthesis of UMOM-1-c

UMOM-1-b was immersed in 36.0 mM dabco solution with 5.0 ml of DMSO/DMF mixture (v/v=1:1) and then left to react at room temperature for 10 min. The reacted crystals were rinsed with 5.0 ml pure DMSO/DMF mixture (v/v=1:1) for three times.

### Synthesis of UMOM-1-d

UMOM-1-c was immersed in 5.0 ml of MeOH and then left to react at room temperature. After 1 day, blue double-shell hollow crystals were collected.

### Characterization

X-ray synchrotron powder diffraction data were taken at the PAL, Republic of Korea. The well ground powder was packed into the 0.5 mm diameter of capillary (wall thickness: 0.01 mm). Diffraction data were collected with ADSC Quantum-210 detector at two-dimensional (2D) supramolecular cyrstallography with a silicon (111) double-crystal monochromator. All data were collected at 298 K with a 150 mm of detector distance using synchrotron radiation (*λ*=1.40009 Å). The ADX programme^51^ was used for data collection and the Fit2D programme (ESRF Internal Report, ESRF98HA01T, FIT2D V9.129 Reference Manual V3.1, 1998) was used for data convert a 2D diffraction image to a one-dimensional diffraction pattern^52^. A single crystal of UMOM-1 coated with paratone-N oil and the diffraction data were collected at 298 K with ADSC Quantum-210 detector at 2D supramolecular cyrstallography with a silicon (111) double-crystal monochromator at the PAL, Republic of Korea. The ADSC Q210 ADX programme^51^ was used for data collection, and HKL3000 (ref. 53) was used for cell refinement, reduction and absorption correction. The single-crystal diffraction data of UMOM-2 and UMOM-1-b′ were collected at 296 K with Mo Kα radiation using a Rigaku R-Axis Rapid II (0.3 mm capillaries with wall thickness: 0.01 mm DMSO solvent). The Rapid Auto software (Rapid Auto software, R-Axis series, Cat. No. 9220B101, Rigaku Corporation) was used for data collection and processing. All crystal structures were solved by the direct method and were refined by full-matrix least-squares calculations using the SHELXL programme package^54^. NMR was performed on an Agilent FT-NMR spectrometer (400 MHz). Ultraviolet–visible absorbance spectra were performed on Agilent Cary 5000. Thermogravimetric Analysis was performed on a TA instrument SDT Q600, heated from 25 to 600 °C under N_2_ atmosphere at a scan rate of 2 °C min^−1^. Gas sorption isotherm was performed on Micromeritics ASAP 2020 instrument. Pore size distributions were obtained using oxide surface cylindrical model with a N_2_ isotherm. Field emission SEM images were obtained from Hitachi S-4800 at an acceleration voltage of 5 kV. FIB-SEM images were obtained from FEI Helios 4850 HP with current 80 pA at 30 kV and the FIB milling was performed with Ga ion beam current of 2.5–9.3 nA at 30 kV.

### Data availability

The X-ray crystallographic coordinates for structures reported in this Article have been deposited at the Cambridge Crystallographic Data Centre (CCDC), under deposition number CCDC 1500392 (UMOM-1), CCDC 1500390 (UMOM-2) and CCDC 1500391 (UMOM-1-b′). These data can be obtained free of charge from The Cambridge Crystallographic Data Centre via www.ccdc.cam.ac.uk/data_request/cif, all remaining data can be obtained from the corresponding authors on request.

## Additional information

**How to cite this article:** Lee, J. *et al*. Evolution of form in metal–organic frameworks. *Nat. Commun.* **8**, 14070 doi: 10.1038/ncomms14070 (2017).

**Publisher's note:** Springer Nature remains neutral with regard to jurisdictional claims in published maps and institutional affiliations.

## Supplementary Material

Supplementary Information Supplementary Figures, Supplementary Tables, Supplementary Methods and Supplementary References

Supplementary Data 1 Single crystal structure for UMOM-1

Supplementary Data 2 Single crystal structure for UMOM-1-b'

Supplementary Data 3 Single crystal structure for UMOM-2

Supplementary Movie 1 Formation of single-shell hollow UMOM-1-b' from UMOM-1 after treating dabco linker for 10 min (x10 speed)

Supplementary Movie 2 Formation of single-shell hollow UMOM-1-b' from UMOM-1 after treating dabco linker for 90 min (x10 speed)

## Figures and Tables

**Figure 1:**
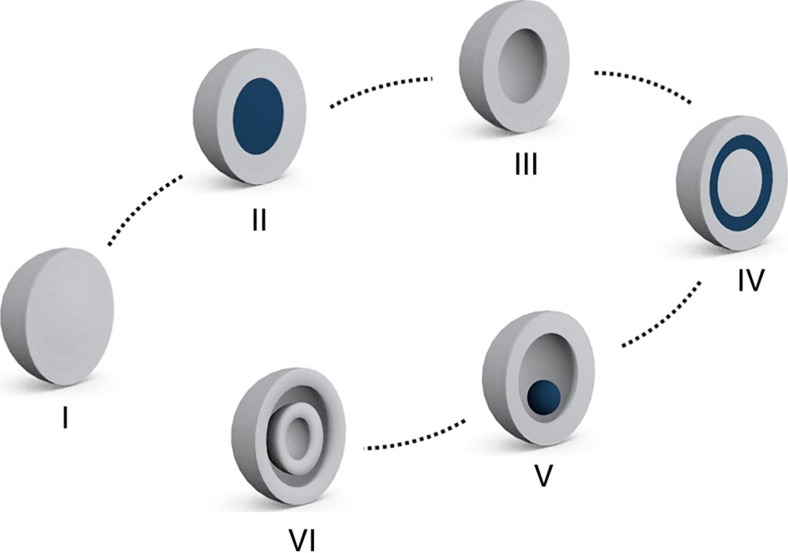
Schematic representation of various forms of micro-/nanostructures. Solid, core-shell, hollow, matryoshka, yolk-shell and multi-shell hollow structures (from I to VI, respectively).
