# Supplementary material for: Evolution of form in metal–organic frameworks
Source: Nat Commun. 2017 Jan 4;8:14070. doi: 10.1038/ncomms14070 (PMC5216133; doi:10.1038/ncomms14070)
Supplement: Supplementary Information — Supplementary Figures, Supplementary Tables, Supplementary Methods and Supplementary References [file ncomms14070-s1.pdf]

Supplementary Figures

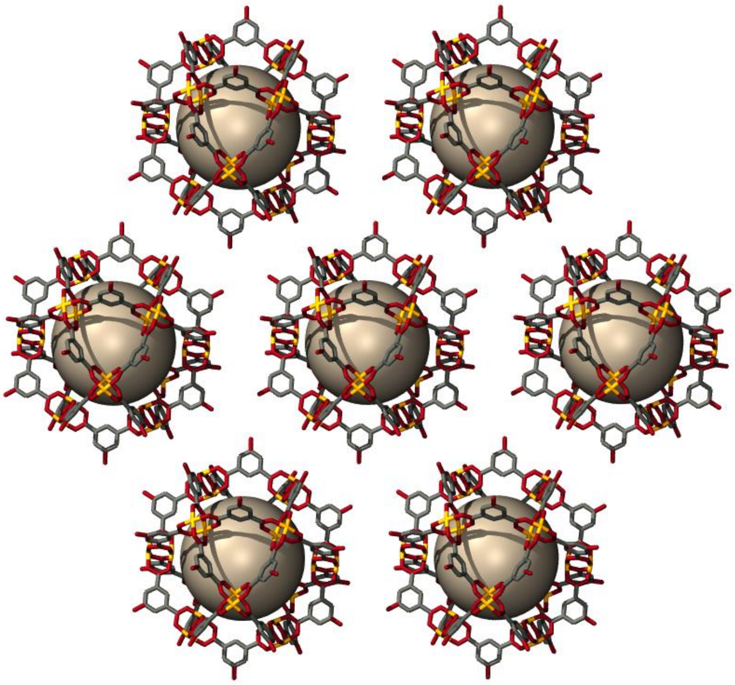

Supplementary Figure 1. Perspective view of the (10-1) plane of the UMOM-1. Cu, orange; C, grey; O, red; all hydrogen atoms, and solvent molecules on the Cu(II) paddle-wheel are omitted for clarity.

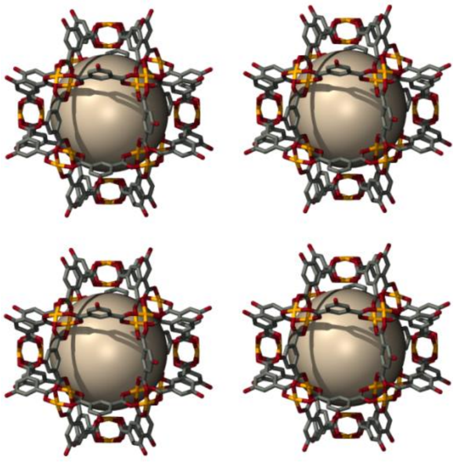

Supplementary Figure 2. Perspective view of the (1-10) plane of the UMOM-1. Cu, orange; C, grey; O, red; all hydrogen atoms, and solvent molecules on the Cu(II) paddle-wheel are omitted for clarity.

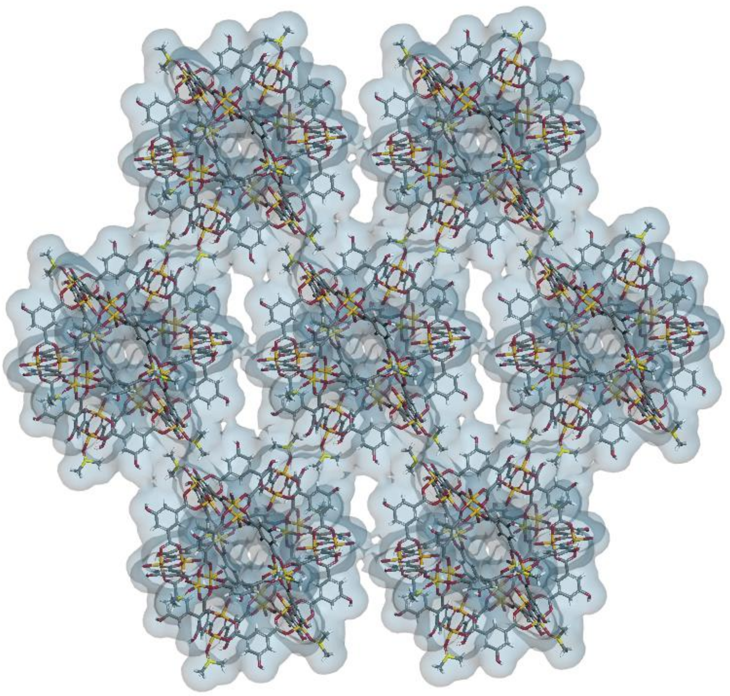

Supplementary Figure 3. Surface of the (10-1) plane of the UMOM-1 with the Connolly surface of 1.4 Å van der waals scale factor using Material Studio.

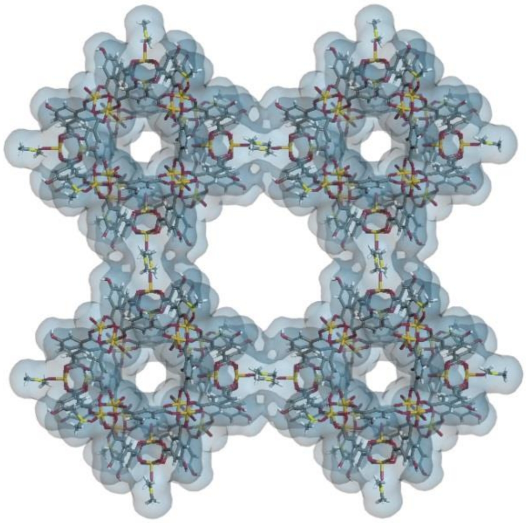

Supplementary Figure 4. Surface of the (1-10) plane of the UMOM-1 with the Connolly surface of 1.4 Å van der waals scale factor using Material Studio.

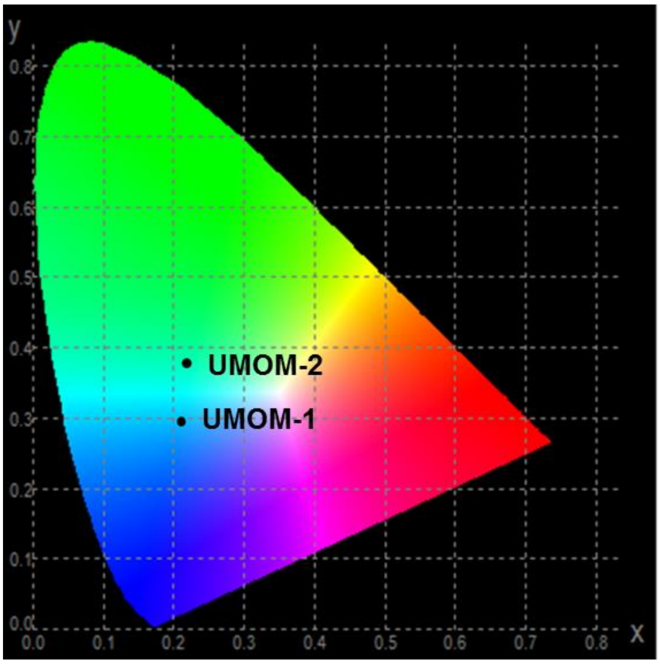

Supplementary Figure 5. CIE diagram showing the color difference between UMOM-1 and UMOM-2 after the single-crystal to single-crystal transformation reaction.

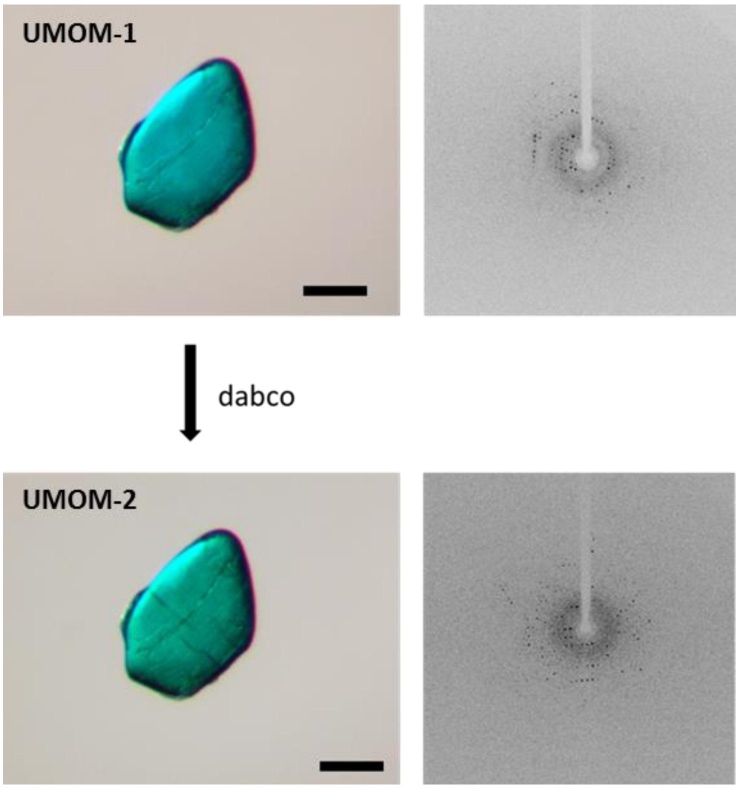

Supplementary Figure 6. Optical microscopic images and single crystal X-ray diffraction images of UMOM-1 before (top) and UMOM-2 after single-crystal to single-crystal transformation (bottom). Single crystal X-ray diffraction images of UMOM-1 as well as UMOM-2 crystal before and after the transformation were the clear evidence for the single-crystal to single-crystal transformation from MOP to MOF.[1] A submicron size of single crystal of UMOM-1 (MOP) was selected and diffracted at 173 K with Mo Kα radiation. The same single crystal of UMOM-1 (MOP) immersed into 0.18 mM of dabco solution overnight. After the reaction, a greenish tinge crystal (MOF) was obtained and diffracted at 173 K with Mo Kα radiation. The scale bar in the optical microscopic images represents 100 μm.

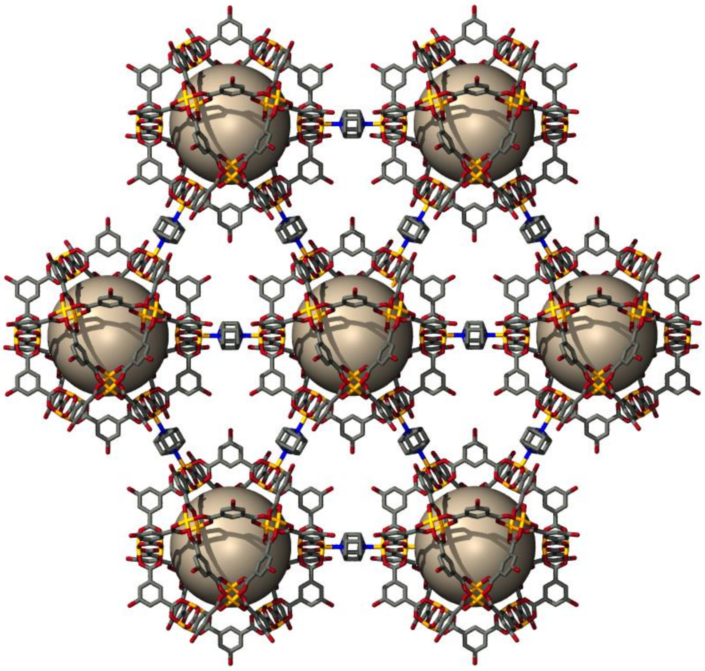

Supplementary Figure 7. Perspective view of the (111) plane of the UMOM-2. Cu, orange; C, grey; O, red; N, blue; all hydrogen atoms, and solvent molecules on the Cu(II) paddle-wheel are omitted for clarity.

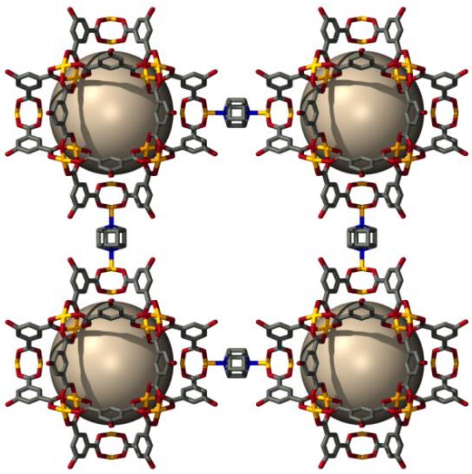

Supplementary Figure 8. Perspective view of the (020) plane of the UMOM-2. Cu, orange; C, grey; O, red; N, blue; all hydrogen atoms, and solvent molecules on the Cu(II) paddle-wheel are omitted for clarity.

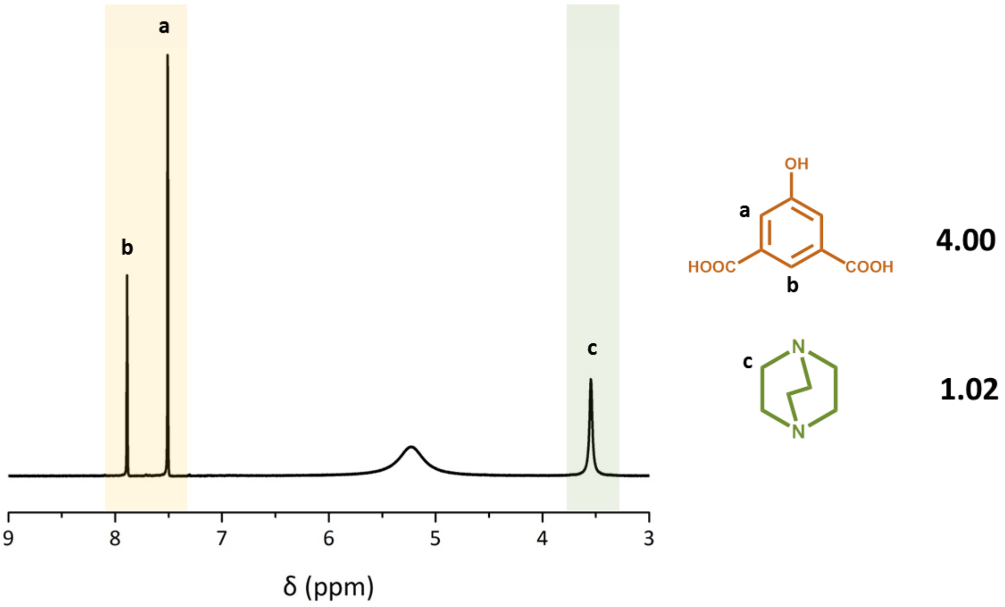

Supplementary Figure 9. ^{1}H-NMR spectrum of UMOM-2 after dabco insertion reaction for 12 h. Approximately 10.0 mg of UMOM-2 crystals were washed with 10mL of DMF/DMSO (v/v=1:1) solution for three times and dried under vacuum overnight and digested the crystals using 0.5 mL of DMSO-d_6 and 0.1 mL of dilute DCl (0.1 mL of 35% DCl in D_2O in 0.5 mL of DMSO-d_6). Orange region represents OH-mBDC linker and green region represents dabco. Residual peak is H_2O (5.23 ppm).

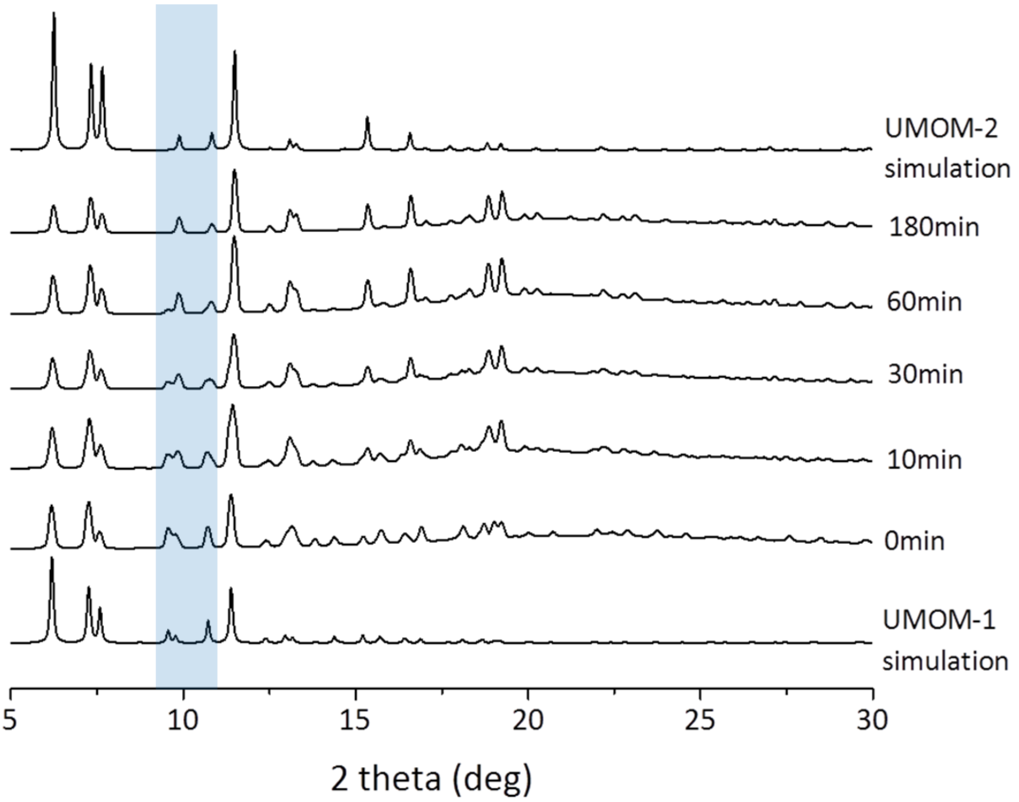

Supplementary Figure 10. X-ray powder diffraction patterns for the time process of transformation experiment. Blue region represents noticeable change of 2θ values at 9.6°, 9.8°, and 10.7°.

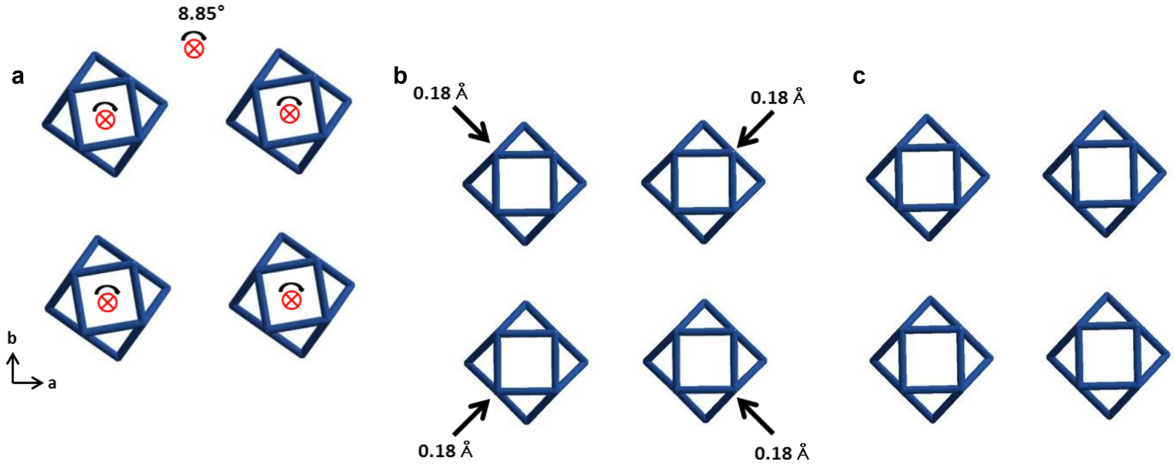

Supplementary Figure 11. A plausible mechanism for structural transformation from UMOM-1 to UMOM-2: (a) Each MOP cages rotate 8.9° along the c-axis. (b) Translation 0.18 Å inward. (c) The result of rotation and translation.

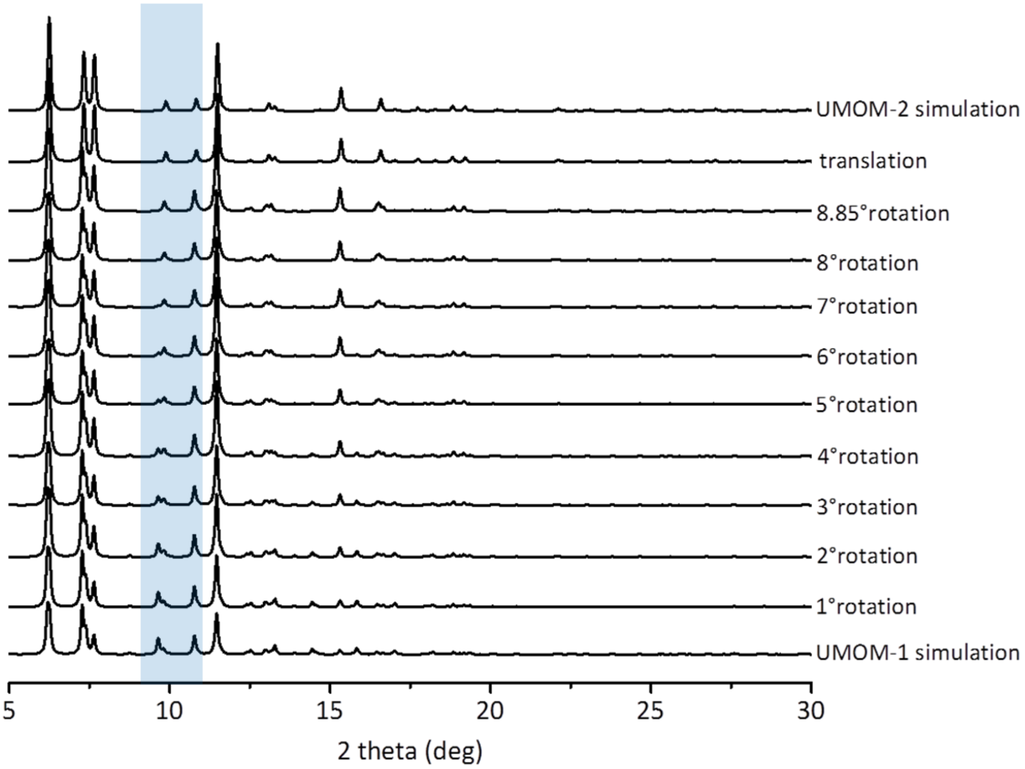

Supplementary Figure 12. The simulation of X-ray powder diffraction patterns for rotation and translation motion during transformation. Blue region represents noticeable change of 2θ values at 9.6°, 9.8°, and 10.7°.

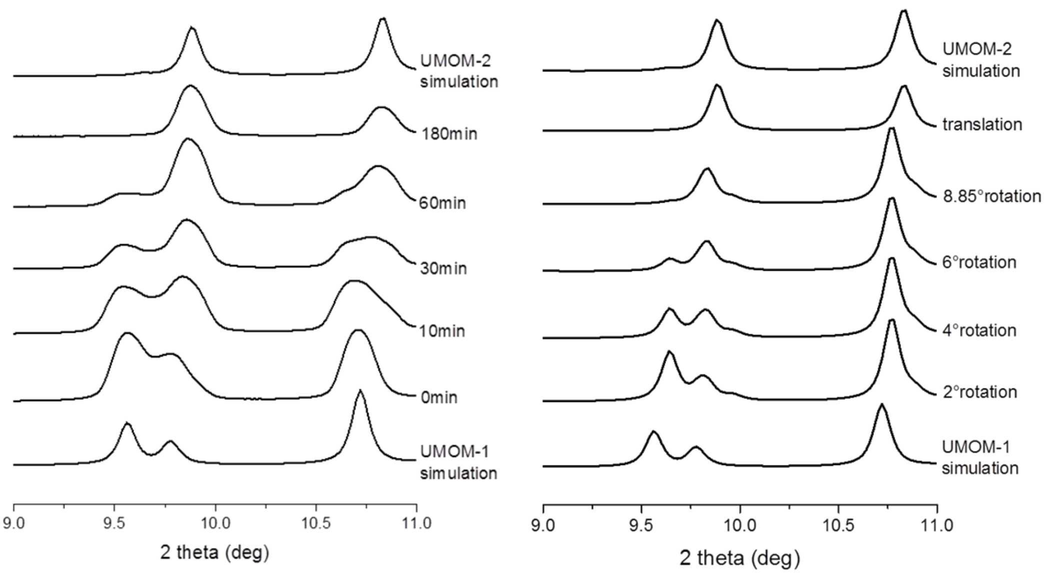

Supplementary Figure 13. Comparing experimental (left) and simulated (right) X-ray powder diffraction patterns.

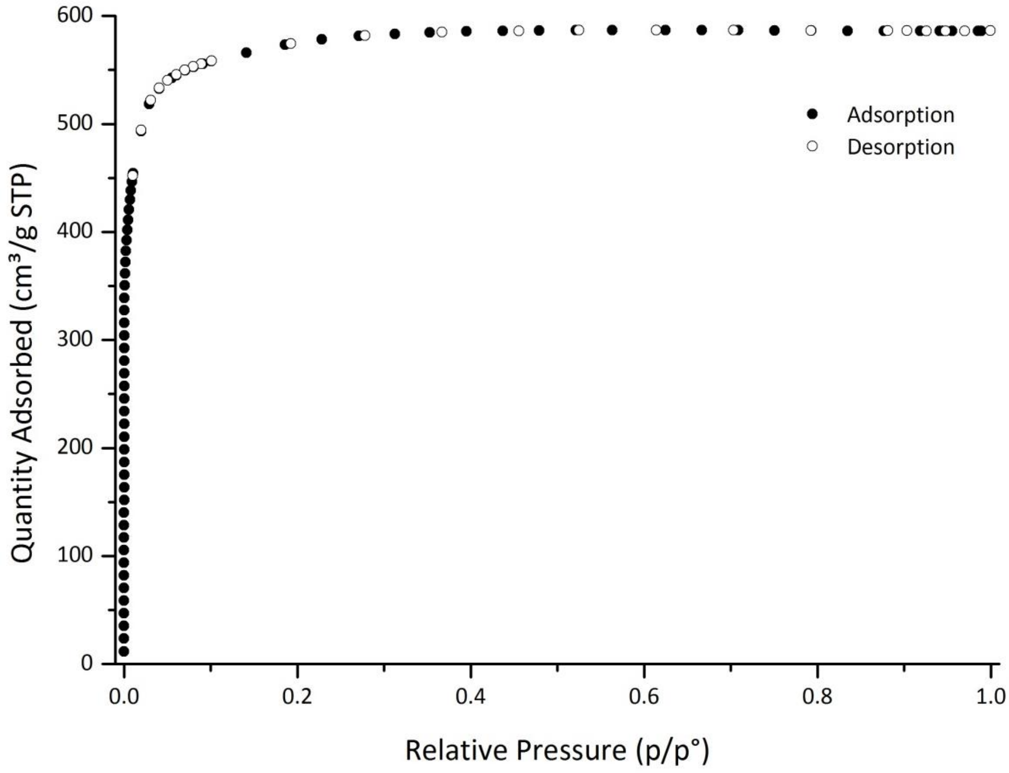

Supplementary Figure 14. N_2 sorption isotherm of UMOM-2 at 77 K after activating at 100 °C for 5 h. BET and Langmuir surface area for UMOM-2 is 2540 and 2820 m^2 g^{-1}, respectively.

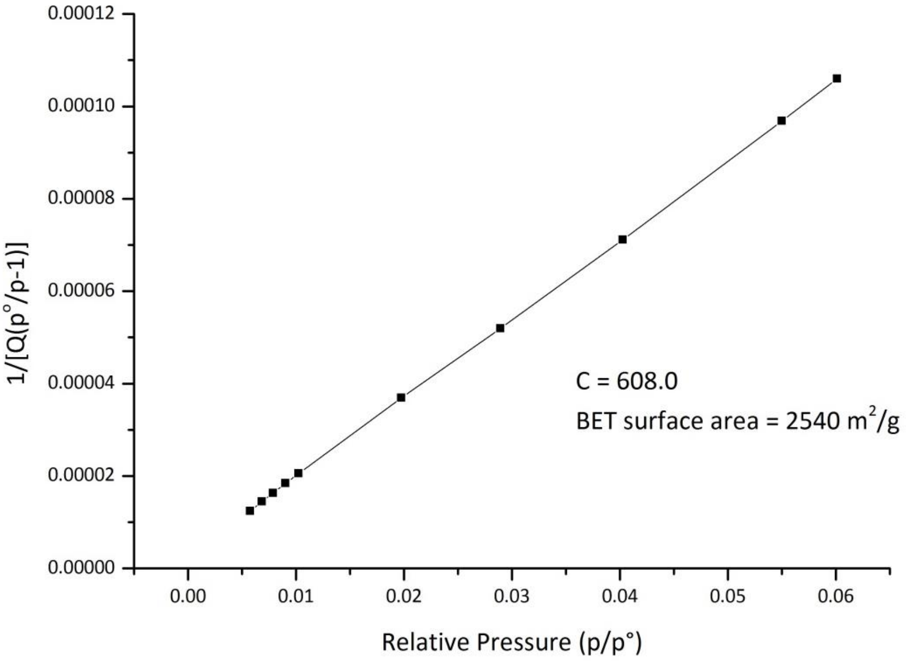

Supplementary Figure 15. BET plot of N_2 sorption data for UMOM-2

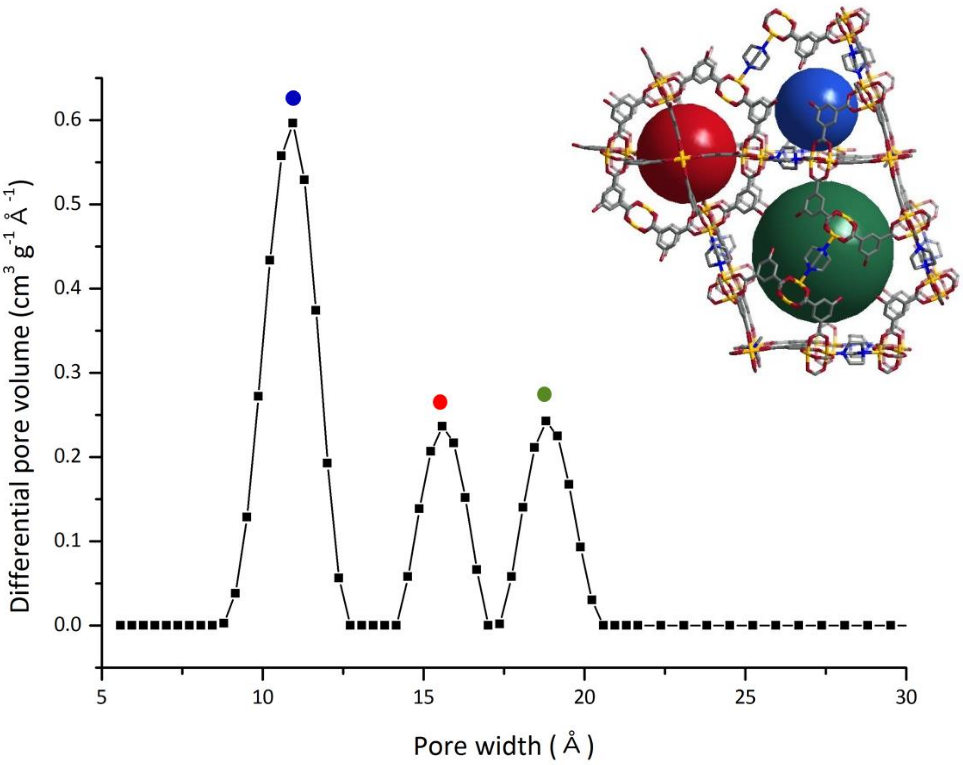

Supplementary Figure 16. Pore size distribution of UMOM-2 measured by the N_2 sorption isotherm at 77 K. Red color represents **cuo**-MOP cage, blue color is truncated tetrahedron cage and green color represents truncated octahedron cage.

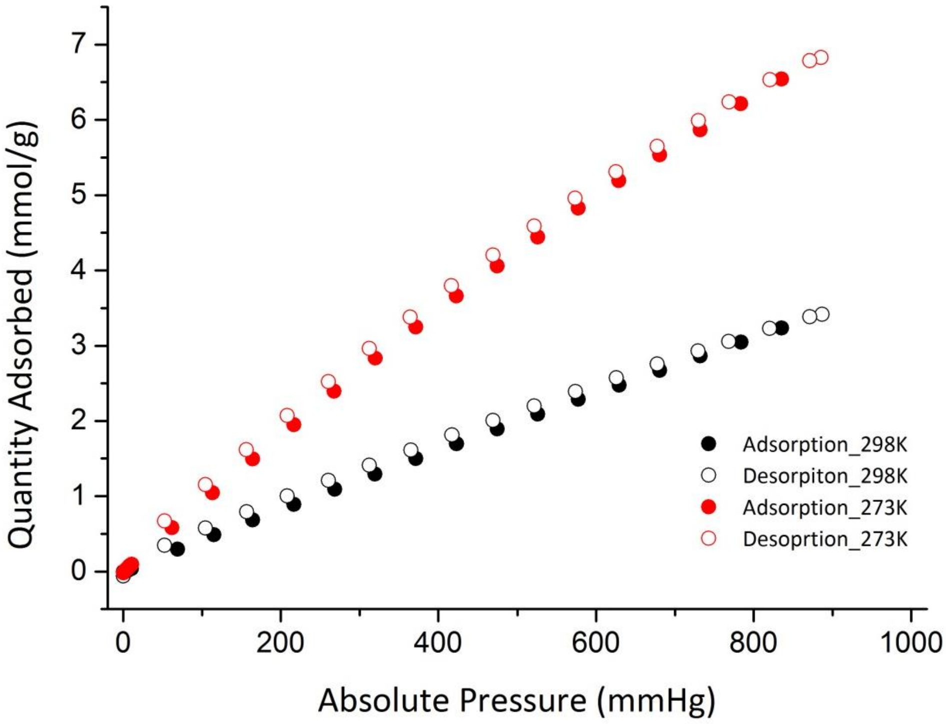

Supplementary Figure 17. CO_2 sorption isotherms for UMOM-2 at 273 K and 298 K.

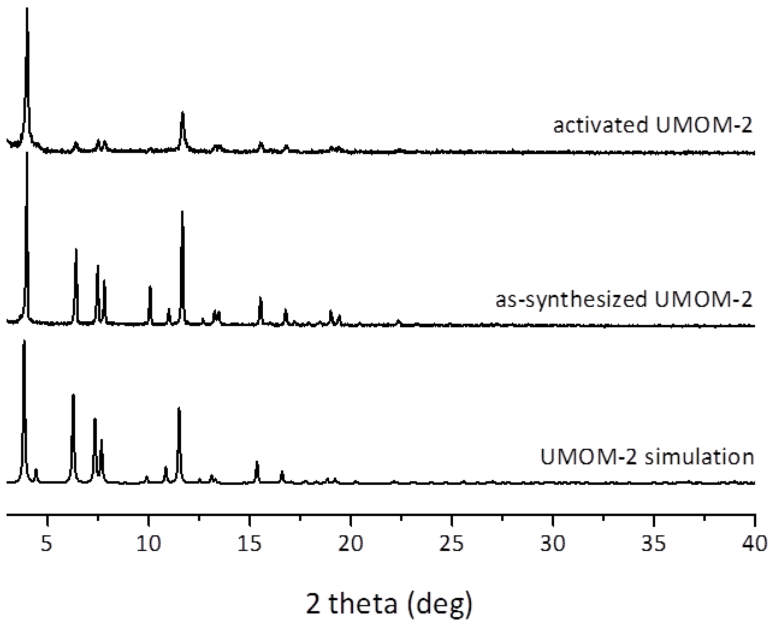

Supplementary Figure 18. X-ray powder diffraction patterns for as-synthesized and activated UMOM-2 at 100 °C for 5 h.

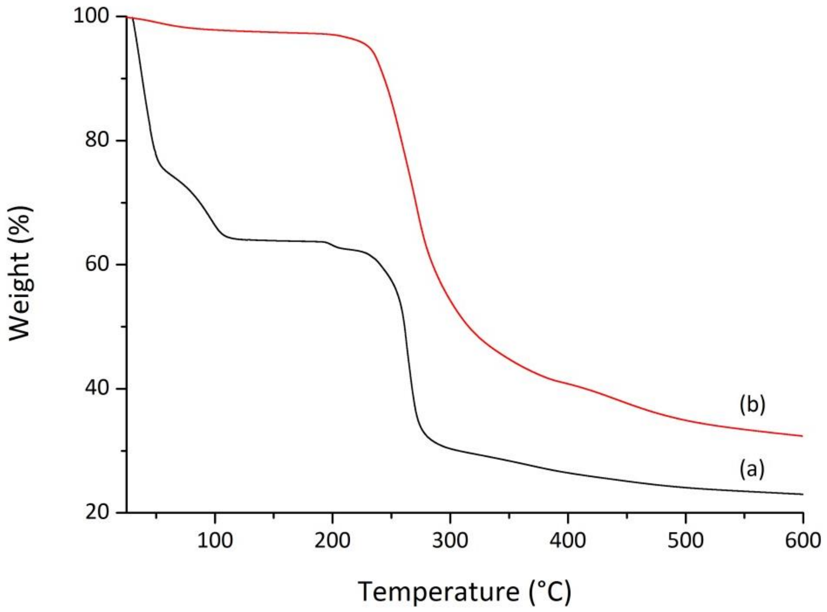

Supplementary Figure 19. Thermogravimetric analysis data for solvent-exchanged UMOM-2 crystals (a) before and (b) after activation process at 100 °C for 5 h. The initial weight loss represents removal of the guest solvent on the crystal surface and in the pore (25-100 °C). The next weight loss around 250 °C represents decomposition of structure.

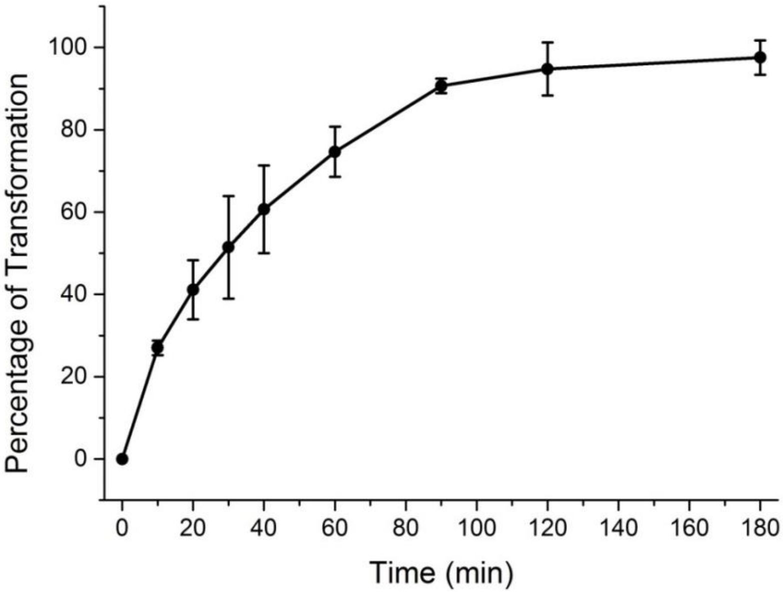

Supplementary Figure 20. Kinetic profile of the transformation procedure based on ^{1}H NMR data from acid-digested solutions of quenched crystals. The digested solutions were prepared by crystals which are collected by quenching the linker insertion reaction at time from 10 min to 180 min. The collected crystals were washed with 10mL of DMF/DMSO (v/v = 1:1) solution for three times and dried under vacuum overnight and digested the crystals using 0.5 mL of DMSO-d_6 and 0.1 mL of dilute DCl (0.1 mL of 35% DCl in D_2O in 0.5 mL of DMSO-d_6). The reaction temperature was kept at 30 °C to exclude the temperature effect on reaction.

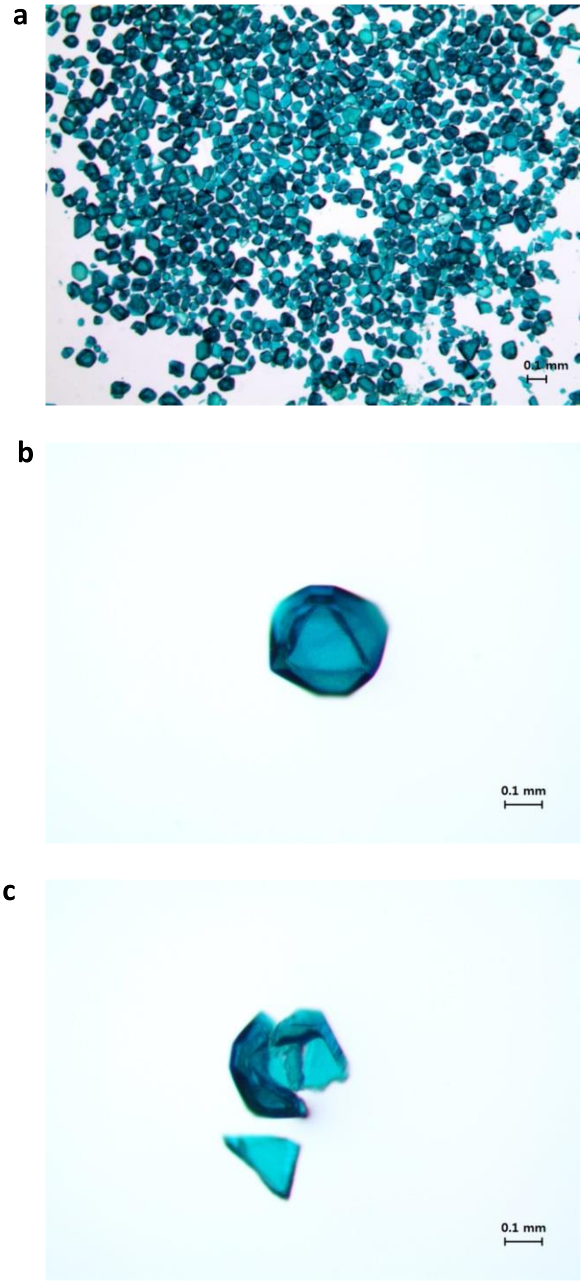

Supplementary Figure 21. Optical microscopic images of (a, b) UMOM-1-b′ and image of (c) broken UMOM-1-b′. Linker insertion time is 90min.

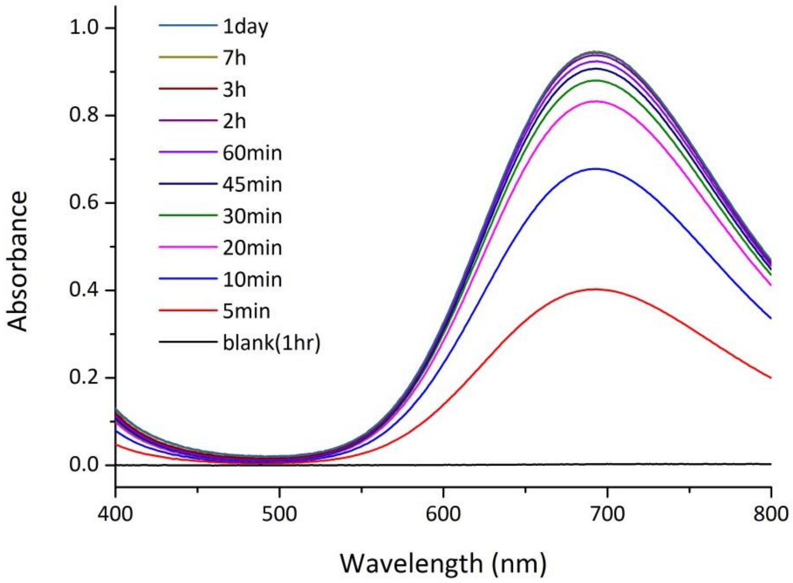

Supplementary Figure 22. UV-visible absorbance spectra of MeOH solution after etching reaction process. The peak at 693 nm represents the existence of Cu(II) ions.[2]

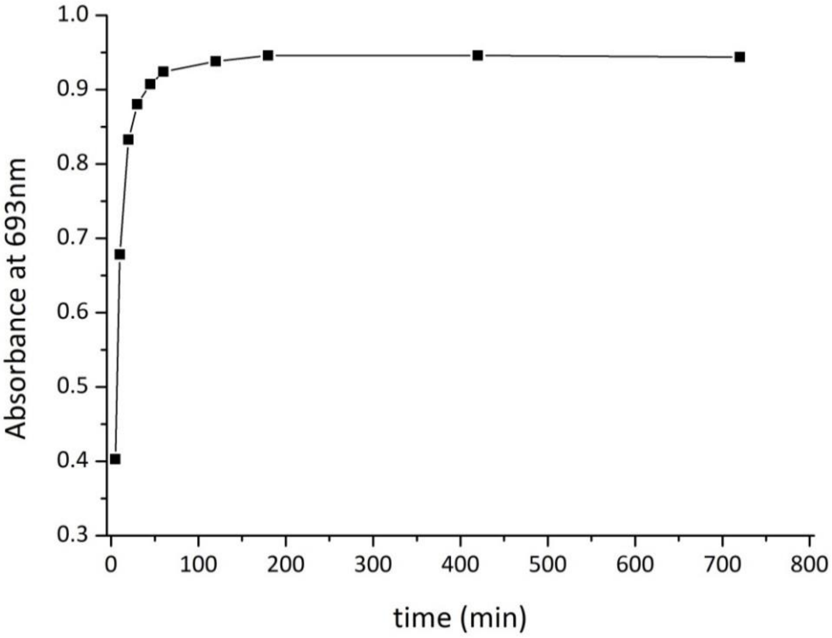

Supplementary Figure 23. The plot of absorbance at 693 nm of MeOH solution which is result of etching reaction process.

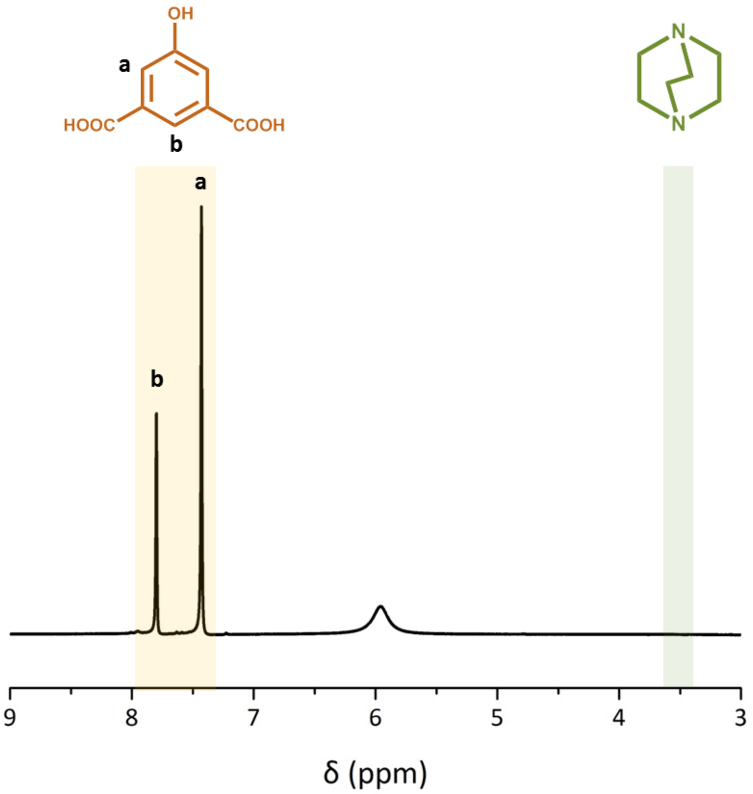

Supplementary Figure 24. ^{1}H-NMR spectrum of digested MeOH solution after 1 day of etching reaction. After evaporate the MeOH solution, result powder was dried under vacuum overnight and digested using 0.5 mL of DMSO-d_6 and 0.1 mL of dilute DCl (0.1 mL of 35 % DCl in D_2O in 0.5 mL of DMSO-d_6). Orange region represents OH-mBDC linker and green region represents dabco.

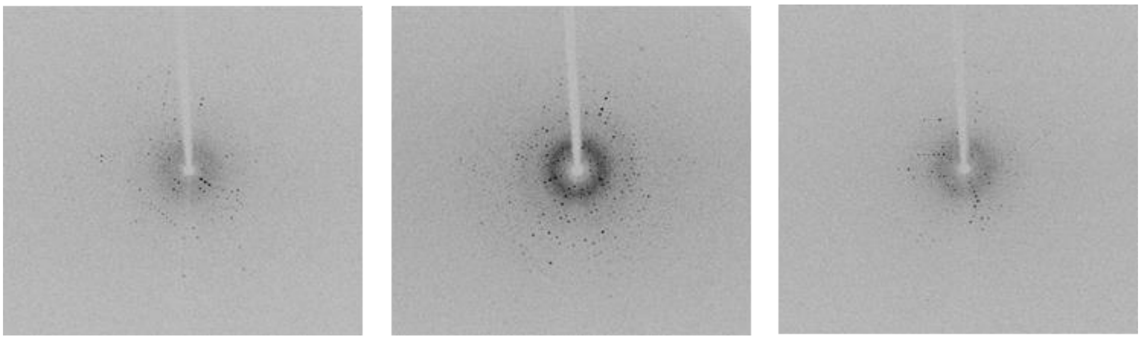

Supplementary Figure 25. Single crystal X-ray diffraction images of UMOM-1-b′ from three crystals. Linker insertion reaction is 10min.

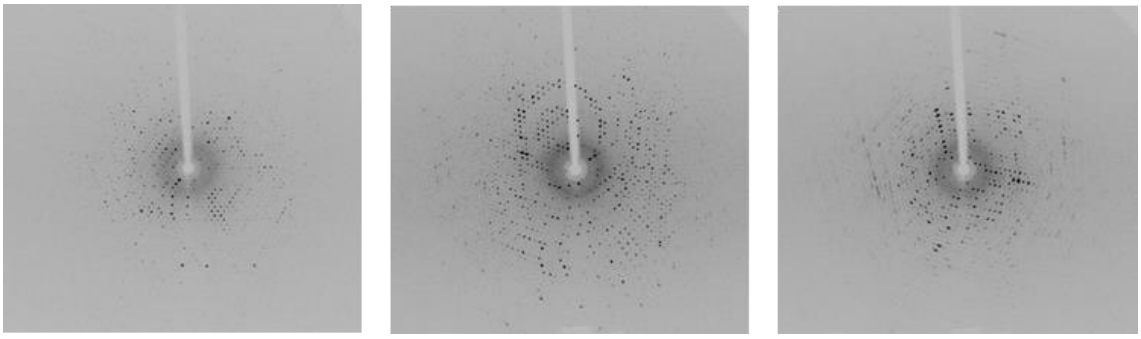

Supplementary Figure 26. Single crystal X-ray diffraction images of UMOM-1-b′ from three crystals. Linker insertion reaction is 90min.

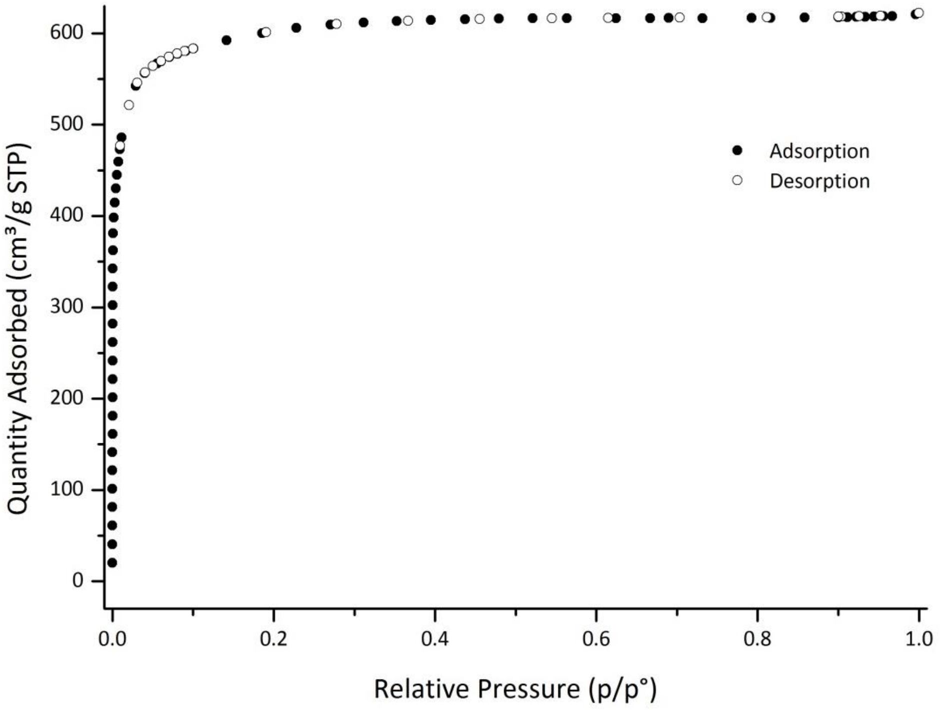

Supplementary Figure 27. N_2 sorption isotherm of UMOM-1-b′, result of 30min linker insertion reaction, at 77 K after activating at 100 °C for 5 h. BET and Langmuir surface area for UMOM-1-b′ is 2390 and 2700 m^2 g^{-1}, respectively.

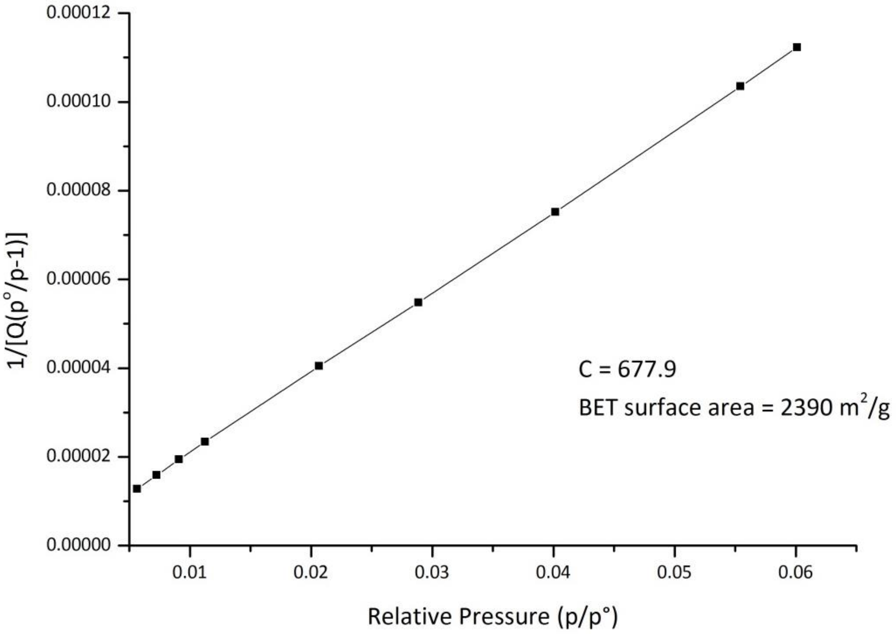

Supplementary Figure 28. BET plot of N_2 sorption data for UMOM-1-b′

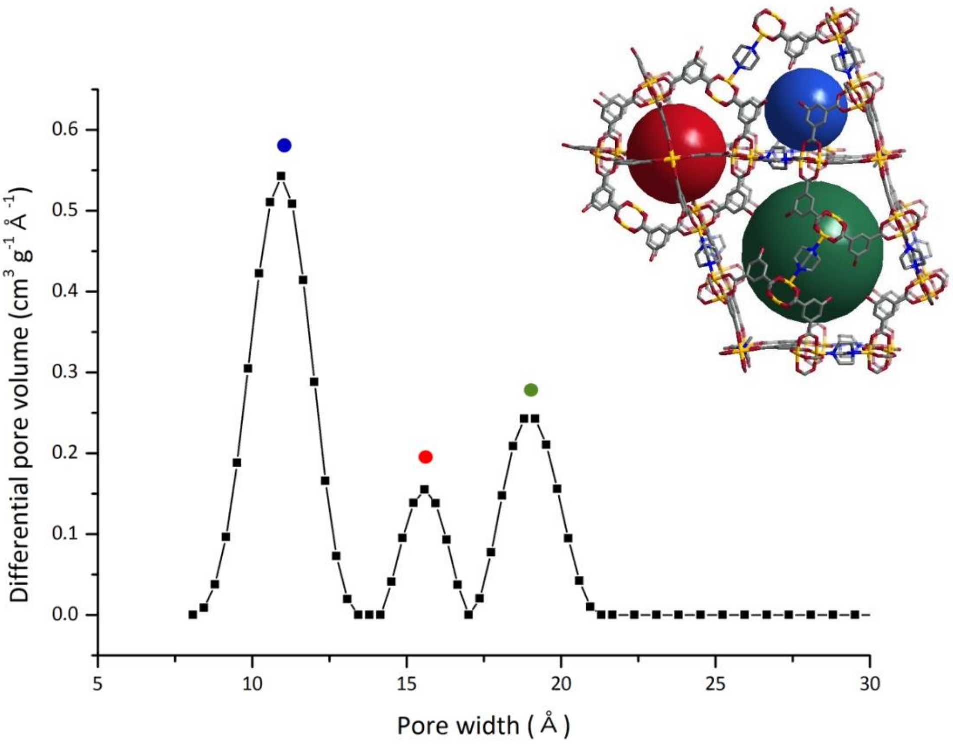

Supplementary Figure 29. Pore size distribution of UMOM-1-b′, result of 30min linker insertion reaction, measured by the N_2 sorption isotherm at 77 K. Red color represents **cuo**-MOP cage, blue color is truncated tetrahedron cage and green color represents truncated octahedron cage.

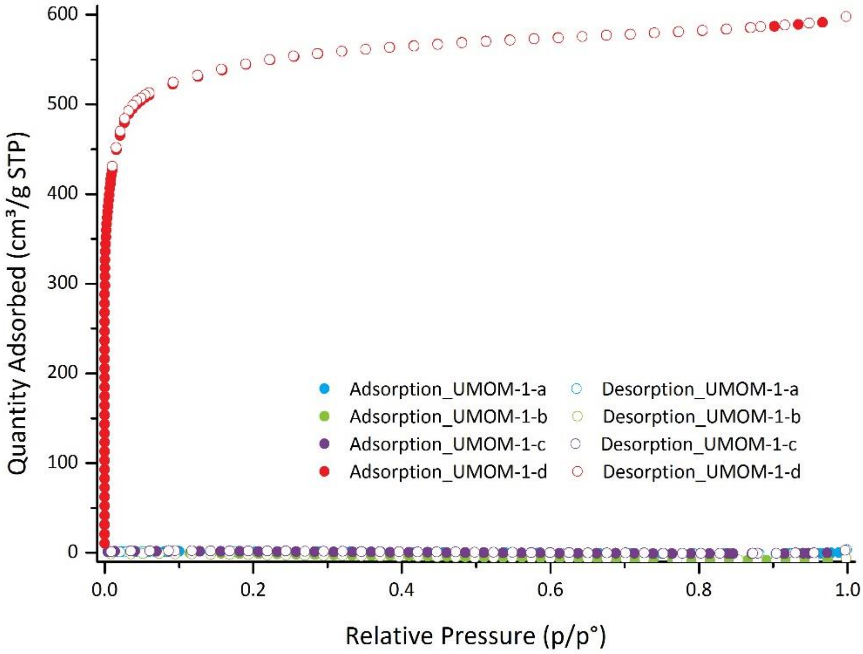

Supplementary Figure 30. N_2 sorption isotherm of various forms of UMOMs, including UMOM-1-a (core-shell), UMOM-1-b (double matryoshka), UMOM-1-c (triple matryoshka) and UMOM-1-d (double-shell hollow). As-synthesized UMOM-1-a, UMOM-1-b, and UMOM-1-c were washed with acetonitrile and UMOM-1-d was washed with methanol three times before the gas sorption experiment. N_2 sorption isotherms of UMOM-1-a, -b, and -c were obtained at 77 K after activating at 130 °C for 3 h and N_2 sorption isotherm of UMOM-1-d were obtained at 77 K after activating at 100 °C for 5 h. BET and Langmuir surface area for UMOM-1-d is 2150 and 2540 m^2 g^{-1}, respectively.

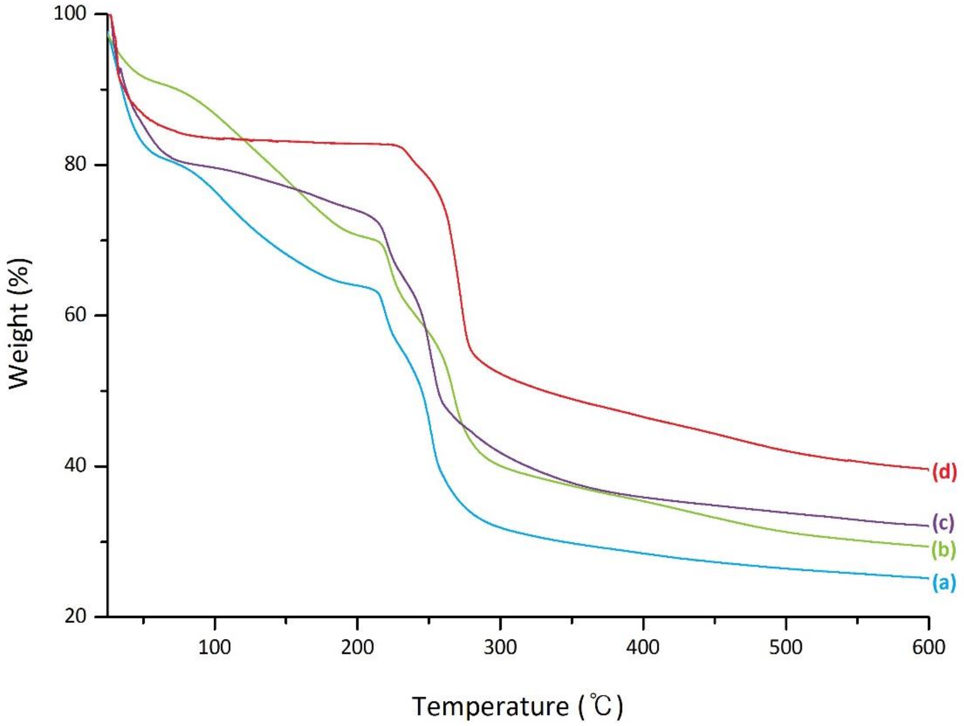

Supplementary Figure 31. Thermogravimetric analysis data for (a) UMOM-1-a, (b) UMOM-1-b, (c) UMOM-1-c, and (d) UMOM-1-d. As-synthesized UMOM-1-a, -b, and -c were rinsed with acetonitrile and UMOM-1-d was rinsed with methanol before the thermogravimetric analysis. The weight loss in the range 25-200 °C represents removal of the solvent molecules in the pore and coordinated the metal nodes. The next weight loss represents decomposition of structure (230-250 °C).

Supplementary Tables

Supplementary Table 1. Known examples of Cu-dabco-Cu distance collected from Cambridge Structure Database.

Ref code	Distance (Å)
XIFWUG	6.6
GUPBEZ	6.7
UMUXAC	6.8
FPDCUO	7.1
NENTIK	7.3
FAGKIH	7.4
XIGLUW	7.5

Supplementary Table 2. Cell parameters of UMOM-1 and -2 before and after the single-crystal to single-crystal transformation

	UMOM-1	UMOM-2
a (Å)	28.23(9)	39.94(9)
b (Å)	28.23(9)	39.94(9)
c (Å)	37.65(7)	39.94(9)
α (°)	90	90
β (°)	90	90
γ (°)	90	90

Supplementary Table 3. Crystal data for UMOM-1

Complex	UMOM-1
Empirical formula	C_{208} H_{168} Cu_{24} O_{144} S_8
Formula weight	6752.86
Crystal system	Tetragonal
Space group	*I4/m*
a (Å)	28.535(4)
b (Å)	28.535(4)
c (Å)	39.508(8)
V (Å^3)	32169(9)
Z	2
ρ_{calc} (g/cm^3)	0.697
μ (mm^{-1})	0.844
R_1, $I > 2\sigma(I)$	0.0877
wR_2, $I > 2\sigma(I)$	0.2814

Supplementary Table 4. Crystal data for UMOM-2

Complex	UMOM-2
Empirical formula	C_{228} H_{192} Cu_{24} O_{132} N_{12}
Formula weight	6736.88
Crystal system	Cubic
Space group	*Fm-3m*
a (Å)	39.954(5)
b (Å)	39.954(5)
c (Å)	39.954(5)
V (Å^3)	63781(13)
Z	4
ρ_{calc} (g/cm^3)	0.702
μ (mm^{-1})	0.825
R_1, $I > 2\sigma(I)$	0.0963
wR_2, $I > 2\sigma(I)$	0.2864

Supplementary Table 5. Crystal data for UMOM-1-b′, result of 90min linker insertion reaction.

Complex	UMOM-1-b′
Empirical formula	$C_{228} H_{192} Cu_{24} N_{12} O_{132}$
Formula weight	6736.88
Crystal system	Cubic
Space group	F m-3m
a (Å)	39.878(5)
b (Å)	39.878(5)
c (Å)	39.878(5)
V (Å^3)	63417(22)
Z	4
ρ_{calc} (g/cm^3)	0.706
μ (mm^{-1})	0.830
R_1, $I > 2\sigma(I)$	0.1486
wR_2, $I > 2\sigma(I)$	0.3738

Supplementary Methods

Synthesis of UMOM-1-d for FIB-SEM images: 2.0 mL of solution-A/MeOH mixture (v/v=1:3) and 2.0 mL of DMSO/DMF solution (v/v=1:1) were carefully mixed. After 2 h, blue crystals were recrystallized. After decanting the solution, the 2.0 mL of 3.6 mM dabco solution was added to the recrystallized crystals and then left to react at room temperature for 15 min. After that, the reacted crystals were rinsed with 5.0 mL pure DMSO/DMF mixture (v/v=1:1) for three times. 2.0 mL of DMSO/DMF mixture (v/v=1:1) and 2.0 mL of solution-A/MeOH mixture (v/v=1:5) were added and left to react at room temperature for 6 h. After decanting the solution, 2.0 mL of DMSO/DMF mixture (v/v=1:1) and 2.0 mL of solution-A/MeOH mixture (v/v=1:5) were added one more and well mixed with crystals then left to react at room temperature for 12 h. The collected crystals were immersed in 3.6 mM dabco solution with 2.0 mL of DMSO/DMF mixture (v/v=1:1) and then left to react at room temperature for 30 min. After that, solvent was exchanged to 3.0 mL of MeOH. After 1 day, blue double-shell hollow crystals were collected.

Supplementary References

1. Assouma, C. D.; Crochet, A.; Chérémond, Y.; Giese, B.; Fromm, K. M., Kinetics of Ion Transport through Supramolecular Channels in Single Crystals. *Angew. Chem. Int. Ed.* ***52***, 4682-4685 (2013).

2. Larsen, R. W. How Fast Do Metal Organic Polyhedra Form in Solution? Kinetics of $[Cu_2(5\text{-}OH\text{-}bdc)_2L_2]_{12}$ Formation in Methanol. *J. Am. Chem. Soc.* **130**, 11246-11247 (2008).
